# Severe pulmonary tuberculosis complicated with insidious pulmonary thromboembolism: a case report and literature review

**DOI:** 10.1007/s11239-019-01967-x

**Published:** 2019-10-13

**Authors:** Lili Huang, Chunyang Yin, Xiaoyan Gu, Xiaojun Tang, Xia Zhang, Chunmei Hu, Wei Chen

**Affiliations:** 1 Department of Tuberculosis, The Second Hospital of Nanjing, Nanjing University of Chinese Medicine, Nanjing, 210003 China; 2 Department of Radiology, The Second Hospital of Nanjing, Nanjing University of Chinese Medicine, Nanjing, 210003 China; 3 Center for Global Health, School of Public Health, Nanjing Medical University, Nanjing, 211166 China; 4 Clinical Research Center, The Second Hospital of Nanjing, Nanjing University of Chinese Medicine, Nanjing, 210003 China

**Keywords:** Pulmonary tuberculosis, Pulmonary thromboembolism, Risk factor, Interventional therapy

## Abstract

Pulmonary thromboembolism (PTE) is an acute and severe disease with high mortality, which is prone to be misdiagnosed or ignored especially when complicated with tuberculosis (TB). Even though TB has been considered as a risk factor for PTE, there is rare report of TB with PTE worldwide. Which TB patients are more susceptible to PTE is still not clear. Here, we described a case report of PTE with pulmonary TB in a 28-year-old man, who had no risk factors for pulmonary thrombosis at admission and developed a medium-high PTE after initiating anti-TB therapy. After local thrombolysis with interventional therapy and sequential intravenous thrombolysis, combined with long-term anticoagulation, the PTE of the patient disappeared. At follow-up of 4 months, the patient was re-examined with chest enhanced CT and no obvious emboli was found. We emphasize that acute or severe TB infection should be included in the thromboembolism risk assessment and prophylactic use of anticoagulants may be considered even if there are no other obvious risk factors. Interventional therapy is a good option for thrombolysis treatment if hospital condition permits.

**Electronic supplementary material:**

The online version of this article (10.1007/s11239-019-01967-x) contains supplementary material, which is available to authorized users.

## Highlights

- Pulmonary thromboembolism (PTE) is an acute and severe disease with high mortality. However, PTE is rarely reported as complication of pulmonary TB.
- No common risk factors for embolization were identified in this TB patient at admission. The occurrence of PTE may be related to severe pulmonary tuberculosis infection, which should be considered as one of the risk factors for PTE.
- Interventional surgery is recommended for local thrombolytic therapy with thrombolysis in TB patients complicated with PTE, if hospital conditions permit.

## Introduction

Tuberculosis (TB) is an ancient and tricky disease caused by *Mycobacterium tuberculous*, which mainly invades human lungs and causes fearful lung dysfunction [1]. Even though many encouraging progresses have been made to control this disease worldwide, it is still difficult to achieve the “Ending TB Strategy milestones” in a short term [2]. At present, TB treatment is challenged by occurrence of multi-drug resistant MTB and complicated by varied comorbidities, including communicable diseases or noncommunicable diseases [3]. Pulmonary thromboembolism (PTE) is a group of noncommunicable diseases or clinical syndromes caused by thromboembolism blockage of pulmonary artery or its branches [4], which is already categorized as the major public health problem, presenting as substantial health-care costs and high mortality [5]. It’s worthy to note that PTE is often associated with varied infectious diseases [6–8]. A population-based case-control study showed that any infections, like pneumonia and symptomatic urinary tract infection, were independent risk factors for PTE [9].

As a chronic infectious disease, TB is also associated with PTE [10, 13]. Further study showed that pulmonary TB induced a systemic hypercoagulable state [11]. The puzzling point is that, however, it’s rare to report PTE as complication of TB. A multicenter registration study found that the annual incidence rate of PTE in China from 1997 to 2008 is approximately 0.1% among hospitalized patients [12]. Among patients with active TB, the prevalence of venous thromboembolism was approximately 2.07% [13]. In addition, some PTEs in TB patients are insidious and develop without specific risk factors and clinical symptoms, causing diagnosis of PTE delayed and missed [14]. Therefore, considering the devasting outcomes, it is crucial and urgent to surveil the indistinctive and ignored clues of the TB patients complicated with PTE through more clinical cases. Here we described an unusual PTE in a non-HIV infective man with very serious TB.

## Clinical case

A 28-year-old man was admitted in our hospital on Jul 13, 2018 because of recurrent cough and yellow-phlegm accompanied by fever for more than 10 months. The symptoms were repeated after routine symptomatic treatment. A chest computed tomography (CT) scan taken in January 2018 had showed that his right lung was spotted by small piece of shadow, accompanied by a cavity. The patient refused to initiate antitubercular therapy. The symptoms were improved after self-administration of cephalosporin as anti-infection treatment for 1 week. In May, the cough and sputum were aggravated, accompanied by fever in the afternoon, with body temperature around 38 °C. In the beginning of July, chest CT showed that the patchy shadow was scattered in two lungs with multiple cavities. The large consolidation in the right upper lung was significantly progressed compared with half a year ago. Considering combination of pulmonary tuberculosis and pulmonary infection, he was hospitalized.

**Fig. 1:**
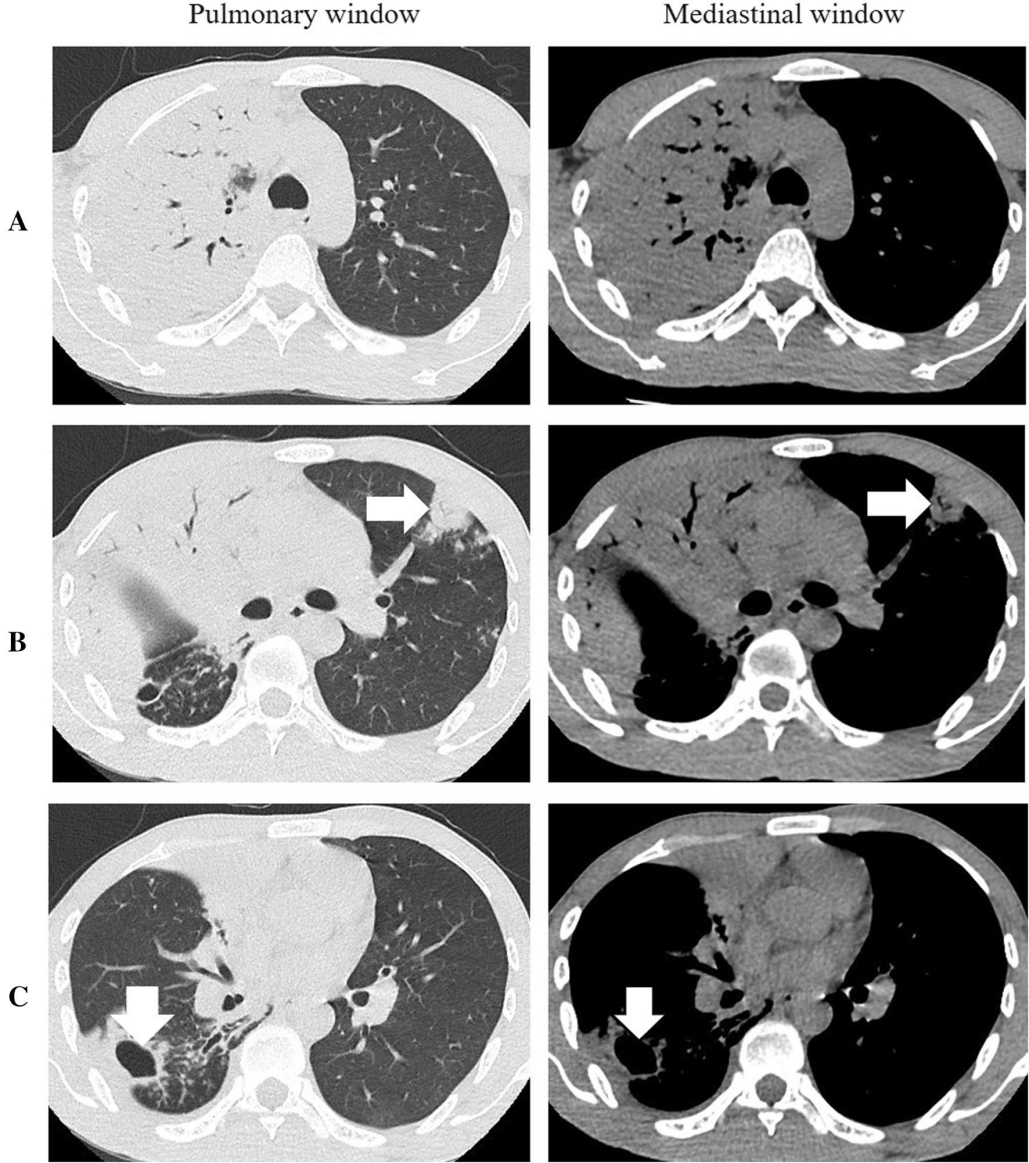
Chest CT confirmed severe pulmonary tuberculosis at admission. Large pieces of density-increased shadow and air bronchogram appeared in the upper right lung (**a**). The small patch of consolidation in the sub-pleura existed in the left lung, where there were small caves indicated by arrow (**b**). In the right lower lung lesion, there was a thin-wall cave, as well as pachynsis and adherence of pleura (**c**). There were neither obvious enlarged lymph nodes in the mediastinum nor the hilar on both sides. The heart shadow was not large, and no effusion or pleural thickening was observed in the chest

At admission, physical examination revealed the following: weight 53 kg; BMI 16.7, body temperature 38.7 °C, pulse 85/min, blood pressure 118/72 mmHg, finger oxygen saturation degree (FOSD) 98%. The lungs had lower breath sounds, with no obvious dry or wet rale. The auxiliary examination showed that the following test items were normal, including blood routine, biochemistry, coagulation, erythrocyte sedimentation rate, arterial blood gas analysis and echocardiography. D-dimer was 13.17 mg/L. Electrocardiogram showed sinus tachycardia. The details of chest CT was shown in Fig. 1. Acid-fast bacilli staining test for sputum sample was positive (4+/3+), which confirmed the diagnosis of pulmonary tuberculosis.

**Fig. 2:**
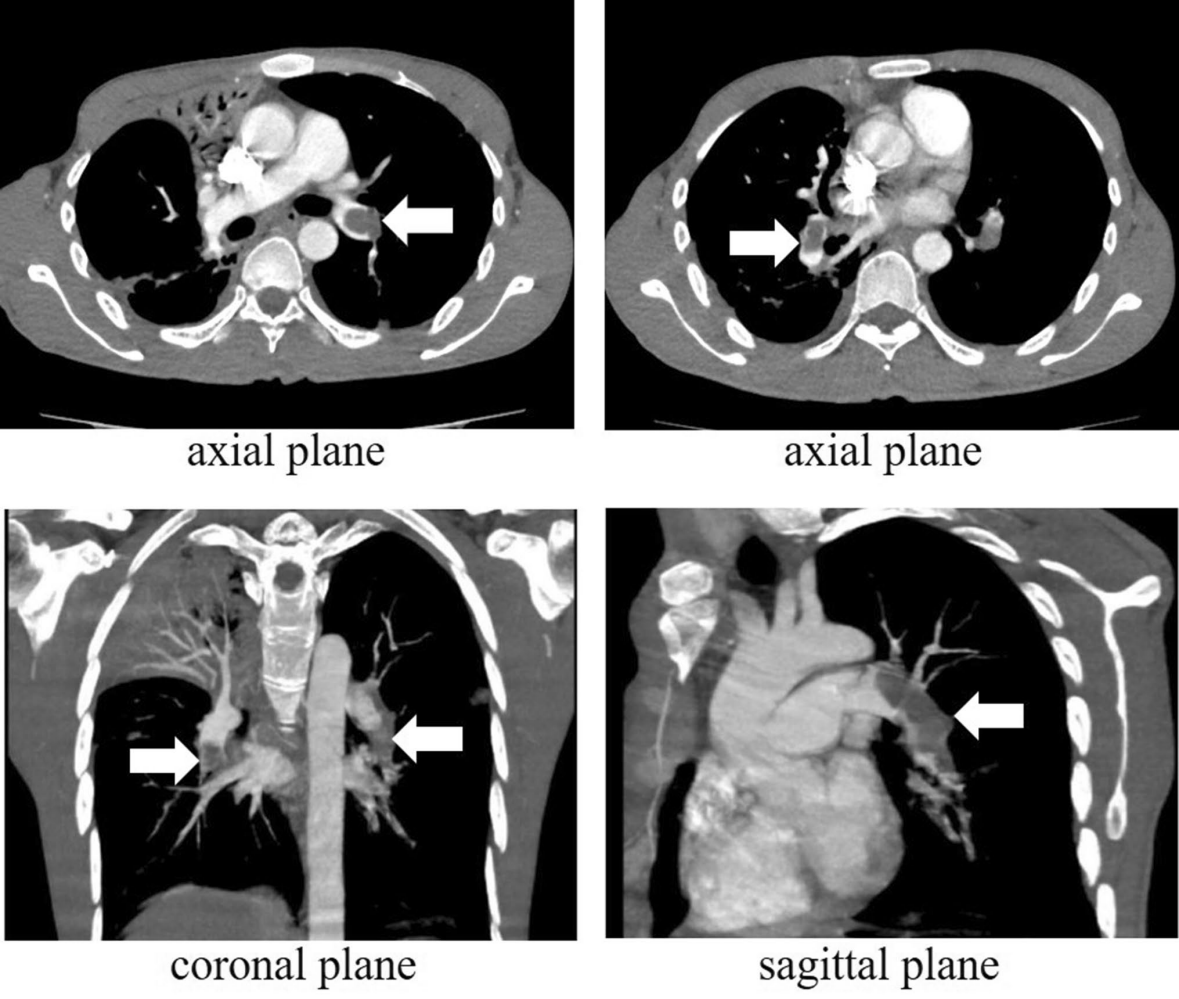
CTPA confirmed PTE after 15 days of treatment. Signs of filling defects were found in the left pulmonary artery, as well as the trunk and branches of right lower pulmonary arteries

Routine anti-tuberculosis regimen was initiated, including isoniazid (300 mg/day), rifampin (450 mg/day), pyrazinamide (1500 mg/day) and ethambutol (750 mg/day). After 3 days of treatment, the body temperature dropped down to the normal, and the cough was relieved. Respiratory symptoms were also improved after 15 days of treatment. At that time, the patient suddenly felt chest tightness, chest pain, and shortness of breath after increased activity (three times more than the usual). Physical examination revealed: heart beats 138/m, blood pressure 88/58 mmHg, FOSD 86%. Even after 6 L high-flow oxygen intake, FOSD was maintained at 90%. Arterial blood gas analysis without oxygen inhalation indicated: pH 7.44, PO2 48 mmHg, PCO2 33 mmHg, SPO2 86%, Oxygenation Index 228.57 mmHg, D-dimer 14.03 mg/L. Electrocardiogram showed atrial tachycardia, non-specific ST elevation. Taking together, all the data highly suggested PTE. Emergency computer tomography pulmonary angiography (CTPA) inspection showed signs of filling defects in the trunk and branches of the left pulmonary artery and the right lower pulmonary artery (Fig. 2).

**Fig. 3:**
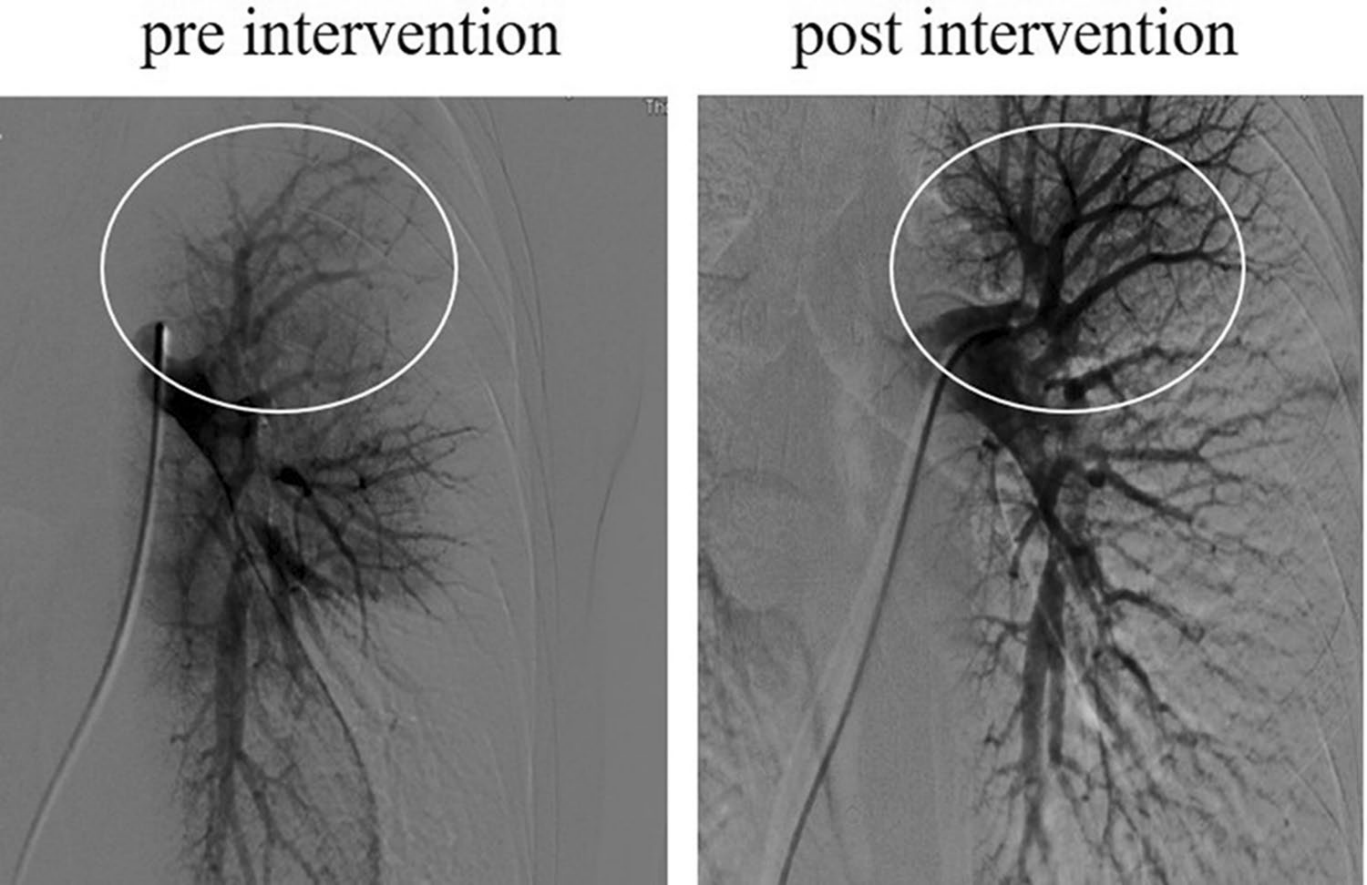
Comparison of pulmonary artery imaging before and after thrombolytic therapy. Pulmonary angiography revealed a filling defect in the left pulmonary artery with poor branch imaging before therapy (left panel). Through a thrombolytic therapy including mechanical thrombolysis, aspiration and pulmonary artery local urokinase thrombolysis, the filling defect of left pulmonary artery disappeared (right panel). The left pulmonary artery trunk and branches were indicated by a circle

According to the CTPA result, the patient was diagnosed as a large-scale PTE, and the risk stratification was medium-high. The patient was prescribed with low-molecular-weight heparin (LMWH) (0.1 mL/10 kg) for anticoagulation. Then, under pulmonary angiography, a combined thrombolytic therapy including mechanical thrombolysis, aspiration, and topical urokinase (300,000 U) was applied to the left pulmonary artery. Fortunately, as shown in Fig. 3, the filling defect of left pulmonary artery disappeared. After the interventional therapy, the patient received intravenous pumping of 20,000 U urokinase every 1 h for 24 h. The symptoms of chest tightness, chest pain and shortness of breath were significantly improved, while the blood oxygen saturation and blood pressure returned to normal, and the vital signs were stable. Neither hemoptysis nor gastrointestinal bleeding occurred during the period of thrombolysis. The deep vein B-ultrasound of double lower limbs was performed and found a flocculent echo in the superior cavity of the upper right femoral vein, and extended into the external iliac vein. Then protein C, protein S activity, coagulation factor V leiden mutation, prothrombin 20210A, lupus anticoagulant, homocysteine, were determined and no abnormality was found. Anti-tuberculosis and warfarin anticoagulant therapy were continued, and international normalized ratio (INR) value was monitored and adjusted between 2 and 3. The patient was discharged with self-administration of oral anti-TB drugs and anticoagulant drug. At follow-up after 20 days of discharge, B-ultrasound showed that the deep vein thrombosis (DVT) of right lower extremity of the patient disappeared. At follow-up of 4 months, the patient was re-examined with chest enhanced CT in the outpatient department of our hospital and no obvious emboli was found.

## Discussion

Even though PTE is the main cause of death from cardiovascular disease [15], PTE is rarely reported as complication of TB. We performed a literature search in Pubmed database with “tuberculosis” and “thromboembolism” as keywords and got only 18 records from 2009 to 2019, which introduced 24 cases of TB complicated with PTE or DVT (summarized in Table S1). These patients were mainly young and middle-aged, with median age 33. Gender was not associated with PTE incidence in TB patients. Besides 2 extrapulmonary TB and 7 undetermined cases, all the remaining 15 cases were severe pulmonary TB, of which, 7 cases were destruction of both lung lobes and complicated with multiple cavities; 3 cases had pleural effusion; 5 cases had underlying diseases, like basal cell carcinoma, diabetes mellitus type II and rheumatoid arthritis. Based on these cases, we inferred that occurrence of thrombosis in TB patients was associated with severity of TB itself, as well as the underlying diseases. Of those 24 thrombosis cases, 15 (62.5%) occurred in pulmonary artery, 13 (54.8%) occurred in deep vein of lower extremity, and 4 (16.7%) occurred in both. Among those 24 patients, 12 (50%) were definitely cured, 2 (8.3%) were dead, and 11 (45.8%) were not recorded for prognosis, suggesting the dangerousness of tuberculosis with PTE/DVT. Oral drug thrombolysis and anticoagulant therapy were the most common treatment methods, except for 1 patient who received mechanical thrombolytic therapy and recovered completely.

It’s known that the three major factors causing thrombosis in TB patients are local lesion invasion, venous compression and hypercoagulability [16]. MTB can directly cause vascular endothelial damage [17] and release chemoattractant such as complement C3a C5a, plasma enzyme activator and kinin releasing enzyme (kallikrein), which can further promote coagulation and thrombosis [18]. Venous compression caused by lymph node tuberculosis could also lead to thrombosis. The occurrence of hypercoagulability was due to elevated blood fibrinogen with impaired fibrinolysis, reduced thrombin III, protein C binding to thrombin, and platelet aggregation [17]. Rifampicin also promoted hypercoagulability, and patients taking rifampicin had 4.5 times higher risk of deep venous thrombosis than those without rifampicin [19]. In addition, because of the enzyme-inducing effect of rifampicin, the dosage of oral anticoagulant must be increased when reaching the therapeutic level of international normalized ratio (INR) [16]. These risk factors are often caused by long-term immobilization or bed rest, which further promote pulmonary embolism.

In our report, the diagnosis of pulmonary tuberculosis was clear. The chest CT indicated a large caseous consolidation in the right upper lung. When the dead cells in cheese tissue were lysed, the products of cleavage and tissue debris could activate the coagulation system to cause embolism and tissue ischemic injury, and the tissue ischemic injury was further aggravated. Furthermore, MTB infection of the patient spanned for almost one year. Long-term chronic infection and hypoxemia increased erythrocyte aggregation, vascular endothelial cells damage, and activation of coagulation factors, which increased coagulation substances and viscosity of the whole blood, resulting in a hypercoagulable state. As a result, DVT of the right lower extremity occurred. When embolus detached and circulated to the pulmonary artery, acute PTE was formed. Actually, even though there are many risk factors for PTE, it is easy to be misdiagnosed or missed due to the poor specificity [20]. At admission, we did notice that D-dimer of the patient was significantly higher than normal and a small patch of consolidation in the sub-pleura existed in the left lung. However, the patient didn’t show any clinical symptoms or signs related to PTE. According to the Pulmonary Embolism Severity Index (PESI) [21], he was considered as PESI class I, which meant the patient was at very low risk of PTE. Therefore, PTE was not be included in the primary diagnosis. In this case we failed to check the B-ultrasonography of lower extremity deep vein during admission examination, and it was not clear whether there was a lower extremity deep vein thrombosis on admission, which was the deficiency in the diagnosis and treatment process of this case.

The key to treat high-risk PTE characterized by hemodynamic disorders is to rapidly dredge the pulmonary artery [22]. Because hypotension means severe pulmonary obstruction, the returned blood volume at left heart is significantly reduced. If this continues, the risk of sudden death would be extremely high [23]. Therefore, the faster the blood vessels are dredged, the more likely it is to avoid serious consequences. At present, there is still no definite conclusion on the treatment of patients with high risk PTE [24]. The major concern in the controversy of thrombolysis for these high-risk patients is how to balance the benefits of treatment and the risk of bleeding. If a treatment is able to effectively improve symptoms without increasing the risk of bleeding or worse, it should be a more reasonable option than anticoagulation. After all, the purpose of anticoagulant therapy is to prevent the spread of thrombus and thrombogenesis, and the dissolution of thrombus depends on the activation of body fibrinolysis [22]. The activation of fibrinolysis in the body has individual differences, and speed is certainly not as fast as thrombolysis. The speed of interventional therapy is not slower than thrombolysis, or even faster and more effective. In addition, the contraindications for interventional therapy are far less than those of thrombolytic therapy in other respects, and the unclear potential bleeding risk during thrombolytic therapy could be avoided. Even though the current guidelines prefer thrombolysis to intervention, it does not mean that thrombolytic therapy is superior to interventional therapy in all aspects. More important reasons lie in the popularity of interventional therapy and the stability of this technology. Once it is mature and stable, interventional therapy is an ideal means.

The computed tomography angiography of this patient was characterized by left pulmonary artery trunk, right inferior pulmonary embolism, hemodynamic instability and decreased finger pulse oxygen, and was diagnosed as a medium -high-risk PTE patient. Therefore, LMWH was prescribed as anticoagulant to promote the dissolution of thrombus by fibrinolytic mechanism. However, there was no significant improvement regarding chest pain, blood pressure and phalanx after medication. Luckily, the interventional department of our hospital possessed the professional technique and condition for PTE interventional therapy, the patient was treated with emergency pulmonary angiography with thrombolysis + aspiration + local urokinase treatment, followed by intravenous pumping of urokinase. There were no thrombolytic and postoperative complications after treatment, and the patients recovered well.

We hereby highlight the risk of severe pulmonary tuberculosis with thromboembolic disease. Prophylactic use of anticoagulants may benefit even if there are no other risk factors for patients with severe or hematogenous disseminated tuberculosis. For medium or high-risk PTE patients, the interventional therapy could be taken into account if conditions permit, in order to obtain the maximum benefit, especially when contraindication or risk of thrombolytic therapy exists.

## Electronic Supplementary Material

Below is the link to the electronic supplementary material

Supplementary material 1 (DOCX 45 kb)
