# Supplementary material for: Severe pulmonary tuberculosis complicated with insidious pulmonary thromboembolism: a case report and literature review
Source: J Thromb Thrombolysis. 2019 Oct 13;49(4):644–50. doi: 10.1007/s11239-019-01967-x (PMC7182625; doi:10.1007/s11239-019-01967-x)
Supplement: Supplementary file 1 — Supplementary material 1 (DOCX 45 kb) [file 11239_2019_1967_MOESM1_ESM.docx]

Table 1. Cases of tuberculosis with PTE/VTE worldwide in last 10 years.

| **Year** | **Type of Tuberculosis** | **Type of** **thrombus** | **Country** | **Age/Sex** | **Underlying disease** | **Treatment** | **Prognosis** | **References** |
| --- | --- | --- | --- | --- | --- | --- | --- | --- |
| 2009 | multiple  cavitary lesions | deep venous thrombosis ,and occlusion of distal segment of left pulmonary artery and right basal segmental branches | Portugal | 31y/male | NA | low-molecular-weight heparin, warfarin and anti-tubercular therapy | NA | [1] |
| 2009 | multiple confluent cavitary lesions | femoropopliteal thrombosis | Portugal | 51y/male | NA | low-molecular-weight heparin, warfarin and anti-tubercular therapy | NA | [1] |
| 2011 | NA | bilateral pulmonary embolism | Poland | 72y/male | basal cell carcinoma | anti-tubercular therapy | NA | [2] |
| 2011 | left-sided pleural effusion | large thrombus in left ileofemoral vein, and thrombus in right pulmonary artery | India | 22y/male | NA | anti-tubercular therapy and oral anticoagulants | recovered fully | [3] |
| 2011 | cervical lymphadenopathy | bilateral pulmonary embolism | India | 35y/male | NA | thrombolized therapy | NA | [3] |
| 2011 | miliary tuberculosis | thrombus in right main pulmonary artery with wedge infarct | India | 40y/male | NA | thrombolized and started on anti-tubercular therapy | recovered fully | [3] |
| 2011 | miliary tuberculosis | thrombus in right main pulmonary artery with wedge infarct | India | 47y/male | diabetes mellitus type II | mechanical breakdown of pulmonary thrombus and thrombolytic therapy followed by anticoagulants | recovered fully | [3] |
| 2011 | pulmoary tuberculosis with right-sided pleural effusion | thrombus in segmental vessels of both pulmonary arteries, right-sided pulmonary infarct, right-sided pleural effusion, superior vena cava thrombus | India | 36y/male | NA | thrombolyzed with urokinase for 24 hours followed by anticoagulation therapy and anti-tubercular therapy | recovered fully | [3] |
| 2014 | extensive bilateral infiltrations and multiple cavitary lesions | bilateral DVT of the common femoral vein | India | 15y/male | NA | anti-tuberculosis therapy and Warfarin | recovered fully | [4] |
| 2014 | multiple bilateral infiltrates with left-sided minimal pleural effusion | thrombus on medial aspect of right femoral vein | India | 13y/male | NA | anti-tuberculosis therapy and Warfarin | recovered fully | [4] |
| 2014 | NA | NA | Tunisia | NA/NA | NA | low-molecular-weight heparin | NA | [5] |
| 2014 | NA | NA | Poland | 31y/male | factor V Leiden mutation | anticoagulants and antibiotics | recovered fully | [6] |
| 2014 | cavitation in the apical lobe of the right lung | bilateral proximal pulmonary emboli | - Cameroon | 52y/male | NA | anti-tuberculosis and anticoagulant therapies | recovered fully | [7] |
| 2014 | nodular opacities of miliary tuberculosis | thrombus within the left axillary and left subclavian veins extending to the proximal left brachiocephalic vein, and pulmonary emboli involving the right lower lobe | - USA | 41y/male | rheumatoid arthritis | anticoagulants with enoxaparin and warfarin | NA | [8] |
| 2015 | cavitary lesion on the right apical region | acute deep vein thrombosis in the left lower limb | Sri Lanka | 37y/female | NA | clarythromycin, enoxaparin, warfarin and anti-tuberculosis drugs | dead | [9] |
| 2015 | NA | thrombosis in the left femoral vein with extension to the infra-renal part of the inferior venacava | India | 11y/female | NA | anticoagulants with low molecular weight heparin and warfarin, and Anti-tuberculosis | NA | [10] |
| 2016 | NA | thrombosis in femoral, superficial, deep femoral and popliteal veins | India | 13y/female | NA | anticoagulants with low molecular weight heparin and warfarin, and anti-tuberculosis | lost to follow-up | [11] |
| 2017 | NA | thrombus in the right pulmonary artery and the right inferior pulmonary vein with a large splenic infarct | India | 31y/male | NA | anticoagulants with low molecular weight heparin and warfarin, and anti-tuberculosis | recovered fully | [12] |
| 2017 | bilateral inhomogeneous infiltrates with cavitation at the right upper zone | DVT in the left pelvic vein, deep femoral vein, external iliac vein, and common iliac vein | India | 23y/female | NA | anticoagulants with low molecular weight heparin and warfarin | recovered fully | [13] |
| 2017 | abdominal tuberculosis | thrombus in posterior lower lobe of sub-segmental branch of the both pulmonary arteries | Malaysia | 22y/female | NA | anticoagulants with heparin and warfarin, and anti-tuberculosis | recovered fully | [14] |
| 2017 | NA | pulmonary embolism in the right lower lobe | Maroc | 54y/male | NA | anticoagulants and anti-tuberculosis | recovered fully | [15] |
| 2017 | a left upper lobe consolidation concerning for tuberculosis | a 3cm by 0.5cm occluding thrombus in the left main pulmonary artery | USA | 33y/female | NA | anti-tuberculosis | dead | [16] |
| 2018 | miliary tuberculosis | pulmonary embolism in the left-ascending pulmonary artery measuring 1.9×0.3 cm | Malaysia | 29y/female | NA | novel anticoagulation therapy with anti- tuberculosis medications | NA | [17] |
| 2019 | multiple cavities lesion in the right upper lobe area | pulmonary venous partial thrombosis in the right upper lobe | USA | 67y/male | hypertension and hyperlipidemia | anticoagulants with low molecular weight heparin and warfarin, and Anti-tuberculosis | NA | [18] |

NA indicated that the full article is not accessed or there is no relative data in the text.

1. Goncalves, I.M., et al., *Tuberculosis and Venous Thromboembolism: a case series.* Cases J, 2009. **2**: p. 9333.

2. Fijalkowska-Morawska, J.B., M. Jagodzinska, and M. Nowicki, *Pulmonary embolism and reactivation of tuberculosis during everolimus therapy in a kidney transplant recipient.* Ann Transplant, 2011. **16**(4): p. 107-10.

3. Mohan, B., et al., *Pulmonary embolism in cases of pulmonary tuberculosis: a unique entity.* Indian J Tuberc, 2011. **58**(2): p. 84-7.

4. Muley, P., et al., *Deep vein thrombosis with tuberculosis: a rate presentation of common disease.* GLOBAL JOURNAL OF MEDICINE AND PUBLIC HEALTH, 2014. **3**(1).

5. Kwas, H., et al., *Pulmonary embolism and tuberculosis.* Asian Cardiovasc Thorac Ann, 2014. **22**(4): p. 487-90.

6. Skowronski, M., et al., *Pulmonary embolism in a young male with tuberculosis and factor V Leiden.* Pneumonol Alergol Pol, 2014. **82**(3): p. 264-70.

7. Ekukwe, N.C., et al., *Bilateral pulmonary embolism in a patient with pulmonary tuberculosis: a rare association in Yaounde, Cameroon.* Pan Afr Med J, 2014. **17**: p. 262.

8. Lee, B. and F. Moosavy, *Pulmonary Embolism following Cessation of Infliximab for Treatment of Miliary Tuberculosis.* Case Rep Pulmonol, 2014. **2014**: p. 479025.

9. Kumarihamy, K.W., D.M. Ralapanawa, and W.A. Jayalath, *A rare complication of pulmonary tuberculosis: a case report.* BMC Res Notes, 2015. **8**: p. 39.

10. Sangani, J., et al., *Tuberculosis and Acute Deep Vein Thrombosis in a Paediatric Case.* J Clin Diagn Res, 2015. **9**(6): p. SD01-2.

11. Gathwala, G., et al., *A Case of Deep Vein Thrombosis and Intracranial Sinus Thrombosis: Possible rare complications of childhood abdominal tuberculosis.* Sultan Qaboos Univ Med J, 2016. **16**(4): p. e516-e519.

12. Bansal, S., K. Utpat, and J.M. Joshi, *Systemic thrombosis due to pulmonary tuberculosis.* Natl Med J India, 2017. **30**(4): p. 201-202.

13. Gupta, A. and R. Dixit, *pulmonary tuberculosis: A neglected risk factor for deep venous thrombosis.* 2017. **6**(2): p. 184-186.

14. Huei, T.J., et al., *A Rare Case of Ileocecal Tuberculosis with Pulmonary Embolism and Deep Vein Thrombosis.* J Clin Diagn Res, 2017. **11**(7): p. Pd03-pd04.

15. Bopaka, R.G., et al., *[Parietal tuberculosis complicated by pulmonary embolism].* Pan Afr Med J, 2017. **27**: p. 107.

16. Ugalde, I., et al., *Fatal Pulmonary Embolism in the Setting of Immune Reconstitution Inflammatory Syndrome Attributed to Ovarian Tuberculosis.* J Investig Med High Impact Case Rep, 2017. **5**(3): p. 2324709617729690.

17. Ahmedy, F., A. Ahmad Fauzi, and J.P. Engkasan, *Asymptomatic tachycardia and acute pulmonary embolism in a case of tuberculosis spondylodiscitis.* Spinal Cord Ser Cases, 2018. **4**: p. 43.

18. Raru, Y., et al., *Pulmonary vein thrombosis secondary to tuberculosis in a non-HIV infected patient.* Respir Med Case Rep, 2019. **26**: p. 91-93.
